# PReS-FINAL-2001: The impact of adalimumab on growth in patients with juvenile idiopathic arthritis

**DOI:** 10.1186/1546-0096-11-S2-O4

**Published:** 2013-12-05

**Authors:** N Ruperto, DJ Lovell, K Jarosova, D Nemcova, V Vargová, H Michels, EC Chalom, N Ilowite, C Wouters, HI Brunner, KK Kracht, H Kupper, E Giannini, A Martini, N Mozaffarian

**Affiliations:** 1 PRINTO-IRCCS, Genova, Italy; 2 PRCSG-Cincinnati Children's Hospital Medical Center, Cincinnati, OH, USA; 3 AbbVie Inc., North Chicago, IL, USA; 4 AbbVie Deutschland GmbH & Co., Ludwigshafen, Germany

## Introduction

Children with juvenile idiopathic arthritis (JIA) often exhibit growth impairments. Treatment with adalimumab (ADA) has been shown to be safe and effective in JIA patients (pts) when dosed every other week (eow) for up to 3 years [1], but the effect of ADA on growth is not known.

## Objectives

The purpose of this post hoc analysis is to describe growth parameters in pts with JIA treated with ADA in a clinical trial setting.

## Methods

Pts aged 4-17 with polyarticular course JIA were enrolled in a phase 3, randomized-withdrawal, double-blind (DB), stratified, parallel-group study, which consisted of a 16-wk open-label (OL) lead-in phase, a 32-wk DB phase, and an OL extension (OLE) phase. In the OLE phase, pts were dosed based on body surface area (24 mg/m2, max 40 mg dose), followed by a switch to 20 or 40 mg eow based on a body weight of ≤30 kg or >30 kg, respectively. To enter the DB phase, pts had to achieve an American College of Rheumatology Pediatric score ≥30% (ACR Pedi 30) during the OL lead-in. Pts could enter the OLE after 32 wks in the DB phase or at time of first flare (whichever came sooner). For this analysis, pts in the DB phase were grouped by baseline weight into 2 groups: ≤33rd percentile and >33rd percentile based on the US Centers for Disease Control and Prevention (CDC) growth charts. All pts who received ≥1 dose of ADA ± methotrexate (MTX) were included in the analysis. Mean CDC percentile changes in height, weight, and body mass index (BMI) percentiles were calculated through 104 weeks. Growth and efficacy data were analyzed using last observation carried forward (LOCF).

## Results

Among the 171 pts enrolled in this study, 144 (84%) met ACR Pedi 30 response criteria at week 16, and 133 (78%) entered the DB phase. Of the 133 pts, 77% were female, with a mean age of 11.2 years, and a mean disease duration of 3.8 years; at baseline, 55 pts (41%) were in the ≤33rd percentile for weight and 78 pts (59%) were >33rd percentile. There were no differences between MTX and non-MTX groups in mean changes from baseline in weight, height, or BMI percentiles (P > .26). Pts in the lower 33rd percentile climbed to a higher mean growth rate through 104 weeks of ADA treatment. For those who started in the >33rd percentile, growth rates showed an initial increase that remained in the normal range throughout the study. Similar patterns were observed for height and BMI percentiles in these 2 groups. ACR Pedi 30/50/70/90 response rates improved over time in both groups, reaching 85%/76%/60%/36% for the ≤33rd percentile group and 83%/76%/51%/29% for the >33rd percentile group by the end of the DB phase with ADA treatment.

## Conclusion

Long-term ADA treatment ± MTX is associated with improvement and maintenance of growth in children with JIA who had experienced impaired development. ADA treatment improved JIA signs and symptoms in both groups, regardless of baseline growth status.

## Disclosure of interest

N. Ruperto Grant/Research Support from: AbbVie Inc., AstraZeneca, Bristol-Myers Squibb, Janssen Biologics B.V., Eli Lilly and Co., "Francesco Angelini", GlaxoSmithKline, Italfarmaco, Novartis, Pfizer, Roche, Sanofi Aventis, Schwarz Biosciences GmbH, Xoma, and Wyeth Pharmaceuticals, Employee of: GASLINI Hospital, Speakers Bureau: Astellas, AstraZeneca, Bristol-Myers Squibb, Italfarmaco, Janssen Biologics B.V., MedImmune, Roche, and Wyeth/Pfizer, D. Lovell Consultant for: AbbVie Inc., AstraZeneca, Centocor, Bristol-Myers Squibb, Pfizer, Regeneron, Hoffman La-Roche, Novartis, UBC, Xoma, and Genentech, Speakers Bureau: Wyeth Pharmaceuticals, K. Jarosova: None declared, D. Nemcova: None declared, V. Vargová: None declared, H. Michels Consultant for: AbbVie Inc., E. Chalom: None declared, N. Ilowite: None declared, C. Wouters: None declared, H. Brunner: None declared, K. Kracht Shareholder of: AbbVie Inc., Employee of: AbbVie Inc., H. Kupper Shareholder of: AbbVie Inc., Employee of: AbbVie Inc., E. Giannini Consultant for: AbbVie Inc., A. Martini Grant/Research Support from: AbbVie Inc., AstraZeneca, Bristol-Myers Squibb, Janssen Biologics B.V., Eli Lilly and Co., "Francesco Angelini", GlaxoSmithKline, Italfarmaco, Novartis, Pfizer, Roche, Sanofi Aventis, Schwarz Biosciences GmbH, Xoma, and Wyeth Pharmaceuticals, Employee of: GASLINI Hospital, Speakers Bureau: Astellas, AstraZeneca, Bristol-Myers Squibb, Italfarmaco, and MedImmune, N. Mozaffarian Employee of: Eli Lilly and Co.
